# Three-dimensional mapping of differential amino acids of human, murine, canine and equine TLR4/MD-2 receptor complexes conferring endotoxic activation by lipid A, antagonism by Eritoran and species-dependent activities of Lipid IVA in the mammalian LPS sensor system

**DOI:** 10.5936/csbj.201305003

**Published:** 2013-08-08

**Authors:** Thomas Scior, Jorge Lozano-Aponte, Vianihuini Figueroa-Vazquez, Julian A. Yunes-Rojas, Ulrich Zähringer, Christian Alexander

**Affiliations:** a Departamento de Farmacia, Benemérita Universidad Autónoma de Puebla, C.P. 72570 Puebla, Pue., Mexico; b Department of Hematology and Laboratory for Cellular Therapy, Instituto Maimonides Investigación Biomédica, Cordoba, Spain; c Departamento de Biotecnología, Tecnológico de Monterrey, Campus Puebla, Mexico; d Division of Immunochemistry, Research Center Borstel, Leibniz-Center for Medicine and Biosciences, Borstel, Germany

**Keywords:** Toll-like receptors, MD-2, lipopolysaccharide, molecular modeling, docking

## Abstract

A literature review concerning the unexpected species differences of the vertebrate innate immune response to lipid IVA was published in *CSBJ* prior to the present computational study to address the unpaired activity-sequence correlation of prototypic *E. coli* -type lipid A and its precursor lipid IVA regarding human, murine, equine and canine species. To this end, their sequences and structures of *hitherto* known Toll-like receptor 4 (TLR4) and myeloid differentiation factor 2 (MD-2) complexes were aligned and their differential side chain patterns studied. If required due to the lack of the corresponding X-ray crystallographic data, three-dimensional models of TLR4/MD-2/ligand complexes were generated using mono and dimeric crystal structures as templates and *in silico* docking of the prototypic ligands lipid A, lipid IVA and Eritoran. All differential amino acids were mapped to pinpoint species dependency on an atomic scale, i.e. the possible concert of mechanistically relevant side chains. In its most abstract and general form the three-dimensional (3D-) models devise a triangular interface or “wedge” where molecular interactions between TLR4, MD-2 and ligand itself take place. This study identifies two areas in the wedge related to either agonism or antagonism reflecting why ligands like lipid IVA can possess a species dependent dual activity. Lipid IVA represents an imperfect (underacylated and backbone-flipped), low affinity ligand of mammalian TLR4/MD-2 complexes. Its specific but weak antagonistic activity in the human system is in particular due to the loss of phosphate attraction in the wedge-shaped region conferred by nonhomologous residue changes when compared to crystal and modeled structures of the corresponding murine and equine TLR4/MD-2 complexes. The counter-TLR4/MD-2 unit was also taken into account since agonist-mediated dimerization in a defined m-shaped complex composed of two TLR4/MD-2/agonist subunits triggers intracellular signaling during the innate immune response to bacterial endotoxin exposure.

## Introduction

**Figure 1:**
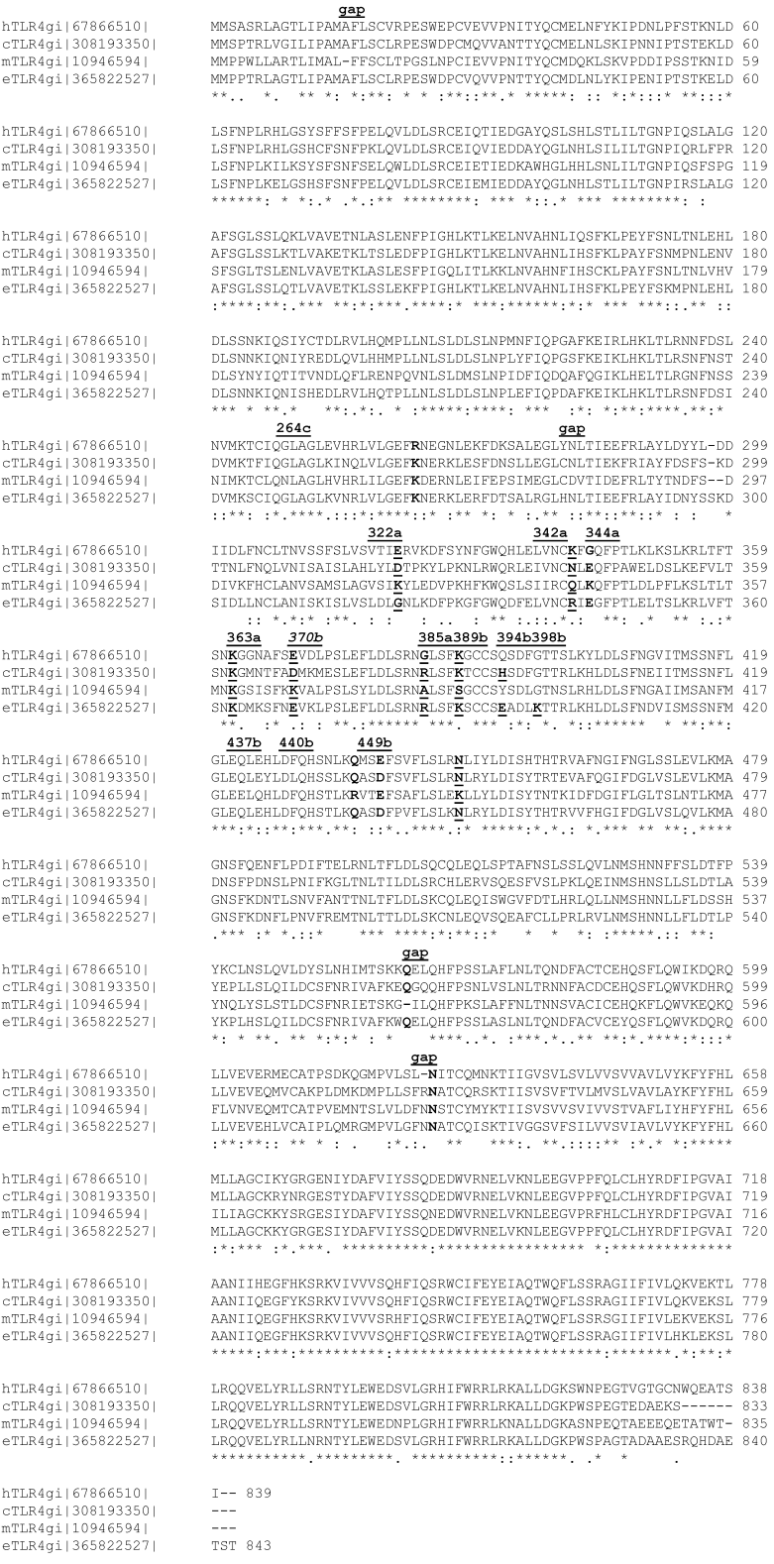
Multiple sequence alignment of the murine, human, equine and canine TLR4 sequences [18, 30]. The TLR4 protein sequence identification number (gi of NCBI records) is given in addition to the species letter (h, c, m or e). A comment line above the sequence blocks refers to residues discussed in the text (underlined, bold face). The last line of a MSA block labels the homology relationship (full identity “*”; high similarity “:”; low similarity “.” while blank space marks missing homology). The residue numbers follow the equine length for being without gaps.

With the advent of crystallography to elucidate membrane protein structures, molecular repositories and software packages, computer simulations have become widely accepted to gain insight into biochemical processes on a molecular level, despite certain setbacks [1–5]. The binding models for liganded TLR4 ectodomain/MD-2 complexes were generated and sequences and proteins aligned (Figure 1 and Figure 2 in [6]). The scope of the present *in silico* study was then to elucidate structural and functional implications regarding the reported species-dependent - either antagonistic or weak agonistic - activity profile of the tetra-acylated ligand Lipid IVA *versus* the species-independent strong immunostimulatory activity of lipid A. Lipid A of the hexa-acylated enterobacerial type represents the central immunoactivating (endotoxic) substructure in lipopolysaccharides (LPS) as characterized for the majority of mammalian commensal Gram-negative bacteria [6, 7]. In comparison to the lipid A and Lipid IVA structures the species-independent TLR4/MD-2 receptor antagonist Eritoran was analyzed in this *in silico* study. In total, structure-activity analyses of a receptor-ligand array composed of TLR4 ectdomain/MD-2 complexes from four mammalian species (man, mouse, horse and dog) and three ligands (lipid A, Lipid IVA and Eritoran) is presented here.

**Figure 2:**
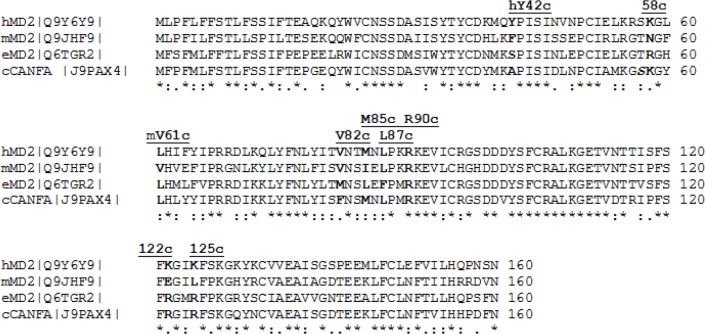
Multiple sequence alignment of the murine, human and equine MD-2 sequences (chain c in 3FXI) [18, 30]. The human sequence was extracted from the crystal structure [18] which served as the 3D template for the mouse and horse models. The canine sequence entry, however, is available as a theoretical prediction. A comment line above the MSA blocks refers to residues discussed in the text (underlined, bold face).

Prior to work the literature was reviewed [6]. *Escherichia coli*-type Lipid IVA activates murine macrophages but antagonizes LPS in human macrophages [7]. It is assumed that such agonistic and antagonistic activity changes for the very same ligand are embedded in TLR4/MD-2 sequence differences among mammalian species [8]. The innate immune system centrally consists of very effective recognition systems for detecting microbial and viral infections on the molecular level: binding of exogenic microbial or viral ligands at minute (picomolar) concentrations to specific immunoreceptors on the cell surface or in intracellular compartments leads to triggering of an effective immune-response [9]. Analytical laboratory work has been driven to the cutting edge of what can be achieved technically concerning isolation, purification and characterization. Due to undetected contaminants when interpreting LPS and congeners activities it matters whether they are obtained from natural sources or *in vitro* synthesis [10–12].

## Methods

In earlier publications, molecular modeling methods were applied and lent mechanistic insights [7, 13–23]. Docking was conducted using Autodock 4.2 following the procedure introduced by Meng *et al*. 2010 [14]. In the following computational simulations it will be shown that ligand binding occurs in the wedge-shaped region between protein units where conserved amino acids and nonhomologous residue changes play a complicated concert of interactions all of which influence the phosphate binding in the interface between TLR4 and MD-2 or in the contact zone of a second TLR4/MD-2 unit leading to dimerization and signaling. Applying ligand docking into a model receptor with automated refinements is a straight forward approach to decipher species differences related to a given binding mechanism when homology between sequences is given and structural templates are known. The rationale for our selection of appropriate software was that Autodock's original calibration set embraces relevant binding patterns for polar (hydrogen bonds, salt bridges) and hydrophobic interactions (alkyl groups) [24–27]. The bibliographic and experimental knowledge gained in earlier docking studies with Insight II, Ludi, FlexX, MOE and Autodock was an invaluable asset during work [4, 5, 28, 29].

### Multiple sequence alignments

In order to visualize the degree of similarity (identity or homology) of amino acid sequences web-based CLUSTAL W [30, 31] was used for multiple sequence alignments (MSA).

### Three-dimensional model generation

Crystal structures were gathered (Table 3 in [6] and [18, 19, 21, 22, 32]) and served as 3D templates [1, 2, 11, 18, 19, 32, 33] for homology protein modeling based on our published experience [34]. The ligands (lipid A, Lipid IVA and Eritoran) were build or extracted from structural data sources (Table 3 in [6] and [18, 19, 21, 22, 32]). The 3D structures of TLR4 and MD-2 models were build as homology models using *Scwrl4* [35], with the same 3D template (PDB code 3FXI [18]).

### Three-dimensional mapping of aligned residues

After multiple sequence alignment studies under *Clustal W* [30] side chain geometries of residues were either kept in case of identities or empirically recalculated into crystal-like conformations by *Scwrl4* [35]. Those residues were highlighted in the alignments and mapped onto the three-dimensional models which are known to be relevant [6]. The dog MD-2 protein has not yet been described experimentally (last visit March 2013, *Universal Protein Resource* at www.uniprot.org). Over a dozen *Sybyl Programing Language* scripts were written to associate the superposed 3D models with published data on important amino acids (FZB licenses during 2009-10) [36]. In the next step the interacting side chains of the computed ligand-protein interfaces were compared with those from our *CSBJ* literature review [6].

### Docking of ligands into the receptor

The initial ligand positions at the binding sites (Table 1) were generated using as main references PDB entries 3FXI (with bound LPS), 2E59 (with bound Lipid IVA), 2Z65 (with bound Eritoran) [18, 19, 21, 32].

**Table 1:** Listing of ligand start positions for docking into the mono or dimeric TLR4/MD-2 complexes. The three species are listed in the first column followed by the ligand type and its start position (initial poses). Cases without data collection are marked by a “-“ sign.

Complex from Species	Initial poses from PDB entry 3FXI	Initial poses copied from 3FXI	Initial poses with flipped backbone	Initial poses from PDB entry 2E59	Initial poses copied from 2E59	Initial poses with flipped backbone
Human	Lipid A	-	Lipid A	-	Lipid A	Lipid A
Murine	-	Lipid A	Lipid A	-	Lipid A	Lipid A
Equine	-	Lipid A	Lipid A	-	Lipid A	Lipid A
Human	-	Lipid IVA	Lipid IVA	Lipid IVA	-	Lipid IVA
Murine	-	Lipid IVA	Lipid IVA	-	Lipid IVA	Lipid IVA
Equine	-	Lipid IVA	Lipid IVA	-	Lipid IVA	Lipid IVA
Human	-	Eritoran	Eritoran	-	Eritoran	Eritoran
Murine	-	Eritoran	Eritoran	-	Eritoran	Eritoran
Equine	-	Eritoran	Eritoran	-	Eritoran	Eritoran
36 starts =	1 +	8 +	9 +	1 +	8 +	9

We manually docked ligands into the unliganded complexes (user-attended docking) and refined interesting parts (glucosamine backbones with phosphate groups) under Autodock 4.2 (unattended docking) [24, 25, 37]. Ligands from known complexes were docked back into their observed poses while all other poses where computed in spatial proximity under the assumption that closely related structures should end up in similar binding modes [18, 19, 21, 32]. Ligand-relevant amino acids of our three-dimensional models were computationally listed for inspection. The ligand's atomic partial charges were calculated by the *Gasteiger* approach under VEGA ZZ [3, 38] while the receptor TLR4/MD-2 complex was prepared under Autodock Tools for docking [37]. The torsion free energies were estimated.

### Docking limitations concerning computed ligand binding into the MD-2 pocket

The established general view is that the observed ligand binding in the crystal complexes is based on hydrophobic interactions and hydrogen bonds on the one hand, and the electrostatic attraction of the phosphate groups on the other hand. This holds true, also, for the models which include fatty acid chains of the LPS and congeners binding deeply into the cleft of MD-2. Their affinities are influenced by noncovalent intermolecular interactions between the two molecules such as hydrogen bonding, electrostatic interactions, hydrophobic and *van der Waals* forces which can be estimated by computational means. Lipoglycans, however, are far from being drug-like, which is the prerequisite for successful application and parametrization of common docking programs like Autodock [24, 37]. Hence, their utility as exploration tools for LPS investigations is fairly limited [4, 5]. The particular challenge of LPS modeling is their pronounced amphiphilic nature: polar parts and ionic centers in addition to vast nonpolar and extremely flexible alkyl chains. Actually, the huge number of rotatable bonds is greatly reduced by coalescence phenomena of the fatty acid segments alone or upon binding in the hydrophobic cleft. The cohesion forces increase with higher fatty acids content and binding depth into the cleft. Hence the entropic quantities (water solvation and dissociation processes, conformational states) of LPS-like ligands must be considered during parametrization since they differ greatly from drug-like ligands. Particularly, by its theoretical nature of the *in silico* approach as introduced by Meng *et al*. 2010 [14] certain assumptions have to be drawn and other implications are related to embeddings under certain methodological operations and working hypotheses expressing awareness about implicit limitations to avoid overinterpretation (Table 2). A critical point in the docking procedure constitutes Autodock's torsional free energy concept (TFE) [24]. TFE is a scaled value and only a crude estimate for torsion entropies. All of which constitutes a serious setback in cases of ligands with extended alkyl scaffold substitutions. TFE values tend to overemphasize the influence of rotatable bonds on acyl chains. With chain length increasing, alkyl chains tend to form a lipid bulk phase (random coil with a droplet-like shape) where much of the rotational freedom is lost. In a similar way, the aliphatic side chains of lipoglycans dock deeply into the hydrophobic binding pocket of MD-2. To this regard, underacylation and shorter acyl chains clearly have an impact. Not only that the hydrophobic contact zone in the MD-2 pocket is greatly reduced but also the tendency of phase coalescences diminishes, i.e. cohesion energies of fatty acids in both lipid pockets and droplets.

**Table 2:** Listing of implied assumptions to formulate valid working hypotheses for the molecular modeling approach.

1	The crystal complexes represent biologically relevant structures and functions, especially the fatty acids tend to form randomly coiled conformations rather than discrete positions (driven by entropy) [2, 39].
2	Missing species data can be completed by computational means [34].
3	Differences in amino acids sequences between species explain the agonist-antagonism dualism.
4	Agonistic behavior of ligand other than LPS/LA may be due to contaminants in traces, i.e. false positive responses in low nonmolar ranges, e,g. Rhodobacter sphaeroides lipid A showed Chinese hamster agonism, but was tested as an murine antagonist and hence may be unreliable [10–13, 16] ****.
5	Agonist binding allows the heterodimerization of TLR4-MD-2-Lig complex.
6	Antagonist binding blocks the heterodimerization of TLR4-MD-2-Lig complex.
7	Docking and scoring show sufficiently responsiveness to reflect species differences in the sequences [14].
8	The torsion free energy can be estimated based on the 2D-connectivity graph of the ligand in a static way.
9	The side chain conformations of nonconserved residues can be repaired during protein homology modeling [35] and rearranged to reflect species differences upon docking [24, 25, 37].
10	The resolution of the crystal structures is sufficient allowing the positional elucidation of tiny electron densities corresponding to ligands’ alkyl chains in the hydrophobic patches of the MD-2 pockets, i.e. discarding artifacts through refinement software [2, 18, 39].
11	The acyl chains appear more deeply buried in the hydrophobic cavity of MD-2 in the case of antagonists like Lipid IVA and Eritoran, than lipid A/LPS [18, 19, 32].
12	The agonist position of lipid A/LPS with its protruding fatty acid FA1 is no artifact forced by crystal packing [18, 21, 39].
13	The reviewed mutation studies show no epiphenomena when associated with observed cell activity results [6].

## Results and Discussion

Prior to the present molecular docking simulations to analyze the species-dependency of Lipid IVA activities, all known crystal structures were inspected and aligned (Figure 2 and Table 3 in [6] and [18, 19, 21, 22, 32]). Since Lipid IVA and Eritoran are structurally closely related to lipid A and LPS (Figure 1 in [6]), it can be assumed that they dock into the TLR4/MD-2 complex in a similar position and orientation as observed in LPS crystal complexes [18]. Hence, we choose the LPS pose as the start conformation for all docking runs concerning agonists and the crystal structural binding site positioning of Eritoran for the flipped orientation of antagonists [19]. In its most abstract and general form the 3D models devise a triangular interface (“wedge”) where molecular interactions with all ligands take place. The resulting differential amino acids of human, murine, equine and canine complexes of the TLR4/MD-2 receptors were identified and documented.

**Table 3:** Protein sequence identities of TLR4 and MD-2 for human, murine, equine and canine species by Clustal W [30]. The TLR4 residue lengths are 839, 835, 843 or 833, respectively and 160 for each MD-2 sequence. However, only a theoretical canine protein MD-2 sequence was found at Universal Protein Resource at www.uniprot.org, last visit March 2013).

Subunit	Subunit	id score
cTLR4	eTLR4	79%
hTLR4	eTLR4	77%
hTLR4	cTLR4	73%
hTLR4	mTLR4	66%
eTLR4	mTLR4	66%
cTLR4	mTLR4	64%
cMD-2 (theoretical)	eMD-2	71%
hMD-2	cMD-2	71%
hMD-2	eMD-2	65%
hMD-2	mMD-2	63%
eMD-2	mMD-2	60%
cMD-2 (theoretical)	mMD-2	65%

### Multiple sequence alignments and three-dimensional structures by homology modeling

As a direct result after aligning full sequences of both proteins, the overall scores of sequence identities can be compared (Table 3). It can be seen that murine sequences show lower similarity when compared to the other mammalian species analyzed in this study. This outcome appears to be a molecular reflection of the mice immune system having evolved to respond to a special set of commensal and environmental bacteria within the context of the behavior, food and anatomic needs of rodents as compared to the three other non-rodent species. Moreover, also when comparing the entire sequences, the human MD-2 and TLR4 proteins are more identical to horse than to mouse (Table 3).

The multiple sequence alignments (MSA) [30], reveal a striking uniqueness of murine residues in certain positions of the MD-2 as well as TLR4 sequences (Figure 1 and Figure 2).

Due to nonidentical deletions over evolutionary time the residue numbers in the sequences differ slightly for equivalent positions: (1) for initial segments up to equine position 297: e = h=c = m+1, i.e. one early gap appears in the murine N-term segment; (2) from equine position 297 to 560: e = m+3 = h+1 = c+1 or h = m+2 = e-1 = c-1. Moreover, “a”, “b”, and “c” are shorthand suffixes to residue numbers in chains TLR4, counter TLR4* and MD-2, respectively, and were taken from 3FXI [18]. For instance, mGlu122c can be found as glutamate labeled E in the murine row at position 122 of chain “c” (the MD-2 protein) in the last MSA block of Figure 2. In the equivalent positions, the human, equine and canine sequences appear more closely interrelated than the corresponding mouse TLR4. The results are in line with published biochemical tests showing that Lipid IVA acts as an agonist in mouse, but as partial (or weak) agonist in horse and as antagonist in human cell test systems while Lipid IVA seems not to be an agonist of canine TLR4 [15, 18–20, 32]. For instance, the highest overall identity score (Table 3) is actually found between the dog and horse TLR4. The residues resembling in relevant sequence positions explain why Lipid IVA seems be an antagonist in both cases [20].

### The conformation of the glucosamine backbone

Literature provides knowledge about the physico-chemical characteristics of ring energy barriers, the preferred conformers, and the reactivity and dipole moments of sugar moieties. For glycosidic bonds, the tendency to suffer hydroxylation is more pronounced under acidic conditions and the bonds tend to resist base attack. The two phosphate ester groups shield any acidic attack (e.g. adjacent acidic side chains of residues) by negative charge repulsion. The dipole moment of the substitution pattern of the diglucosamine backbone was investigated to rule out any intramolecular dipolar repulsion effect since it is known to exist, for instance in the case of trans-1,2-dibromocyclohexane which is present in equatorial conformers in polar solvents, that means under dipole reinforcing environment. But, when exposed to nonpolar solvents, the same compound was reported to show both substituents in axial orientation and the dipole effect was canceled. Like their scaffold cyclohexane, monosaccharides and their derivatives predominantly adopt a chair conformation. On theoretical grounds chair-flipping (fast interconversation at room temperature) of the unsubstituted diglucosamine backbone is possible to invert the axial and equatorial positions since the energy barriers of the cyclohexane scaffold are about 10 kcal/mol which can be overcome in a thermal bath at room temperature (threshold 10 to 20 Kcal/mol). But in the case of LPS due to numerous substitutions the chair flipping which has been experimentally observed for cyclohexane at room temperature should be greatly hindered and the resulting conformation with equatorial substitutions becomes predominant in practical terms (with an additive energy gain of 5 kcal/mol for a bulky substituent) [40, 41].

The normal and flipped orientation which was observed in liganded complexes was also dealt with during docking, see the study design in the Methods section (Table 1). When back docking ligand poses which can be observed in 3FXI [18] or 2E59 [32] Autodock could occasionally restore the proper orientation but not in the most populated RMSD clusters (i.e. groups of geometrically similar conformations) of final docked solutions.

### The observed orientation of the glucosamine backbone

In Table 4, the activity-dependent binding patterns become obvious. The ligands are not exactly symmetrical when referring to their common diglucosamine backbone because one phosphate group is in equatorial (the ester-bound P2 of GlcN2) and the other in axial position (the α-glycosidic P1 of GlcN1), which is an important matter influencing their height above the MD-2 opening and their subunit linker capacity for complex dimerization. Apparently only in the “normal” agonistic orientation the phosphate groups can bridge and grasp both TLR4 subunits with sufficiently strong nonbonded forces to side chains of TLR4 and counter TLR4*. Since the activity is not clear, the comparison with 3MU3 remains inconclusive (Table 4 in [6] and [18, 21, 32]).

**Table 4:** Observed binding patterns for ligands and their phosphate groups. P1 (P2) is the glycosidic (ester)-bound HPO_4_^-^ on glucosamine GlcN1 (GlcN2) of the ligand backbone.

Effector activity	Complex	Orientation	Phosphate binding according to Figure 3 in [6].	Ref and PDB Code
Agonist	(hTLR4/hMD-2/LPS)_2_	“normal”	P1 in Pag (uppermost corner of the wedge);	[18]
			P2 in Pag-Pan (rightmost corner of the wedge).	3FXI
Antagonist	(hTLR4/hMD-2/LipidIVa)	flipped	P2 in Pan (leftmost corner of the wedge);	[32]
			P1 in Pag-Pan (rightmost corner of the wedge).	2E59
Antagonist	(hMD-2/Eritoran)	flipped	P2 in Pan (leftmost corner of the wedge);	[19]
			P1 in Pag-Pan (rightmost corner of the wedge).	2Z65
Unclear	(chicken MD-1/LipidIVa)	“normal”	P1 near Pag (uppermost corner of the wedge);	[22]
			P2 near Pag-Pan (rightmost corner of the wedge).	3MU3

### The observed ligand binding into the MD-2 pocket

**Figure 3:**
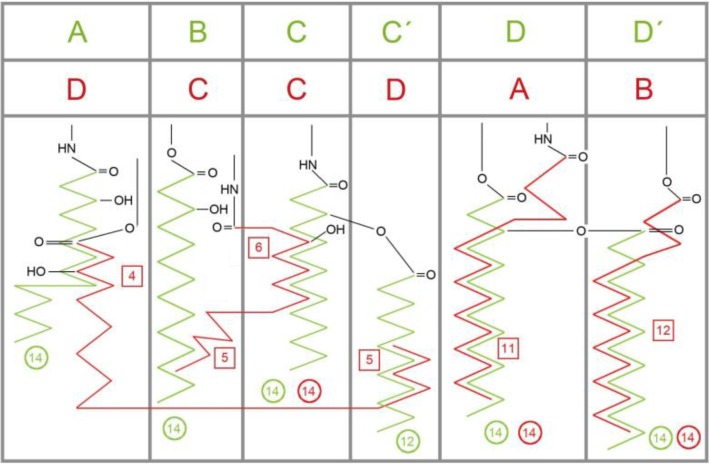
Schematic display of the rotated (′flipped′) cavity occupation in human MD-2 by acyl side chains of agonistic LPS (green) and antagonistic Lipid IVA (red), found in two crystal structures (PDB codes: 3FXI, 2E59) [18, 32]. Cycles give the chain lengths. For instance, in LPS (green) four chains of (R)-3-hydroxytetradecanoic acid (green circles “14”) are attached to the backbone by either amide or ester bonds in positions A, B, C and D which corresponds to R2′, R2′′, R3′ and R3′′ in an alternative labeling convention [18] and the ′secondary′ lauryl (C12) and myristoyl (C14) and residues are mapped in positions C′and D′, respectively. In Lipid IVA (red) there are only 4 chains, labeled A to D. Its chain D partially occupies the corresponding space of LPS chains A and C′ (green). The square boxes indicate the number of carbon atoms in a chain which are overlapping with an adjacent chain (superposition length). The side chains C and D of antagonists (red) are more deeply buried (see the relative location of the amide and ester head groups).

The variable pocket sizes between MD-1 and MD-2 were discussed in the crystallographic literature [14, 32]. While Yoon *et al*. observed an increase in pocket size for chicken MD-1 upon Lipid IVA binding, Ohto *et al*. reported no significant changes when Lipid IVA binds to human MD-2 [32]. The pocket of hMD-2 filled with an agonistic LPS molecule becomes somewhat larger than when occupied by antagonists like Lipid IVA or Eritoran which share a common binding mode with a horizontally flipped backbone compared to LPS due to side chain arrangements [8, 18, 32]. The alkyl chains are superposed for comparison in Figure 3.

### Docking the ligands into the complexes to determine species-dependencies

In order to elucidate the reasons for the backbone flipping, docking simulations were carried out with ligands in observed and flipped starting poses (Table 5). Observed poses are taken from the cases of Lipid IVA and Eritoran. Rejecting docked poses was the result of bad *Van der Waals* contacts, i.e. atomic repulsion forces due to the lack of space to accommodate the ligand in the cavity at the binding site (Table 5).

**Table 5:** Accepted or rejected poses after attended docking of ligands by *van der Waals* (vdW) forces. Listing of agonist (ag) or antagonist (an) poses according to x-ray data (x) or inverted (i) backbone (BB) orientation. The crystal complex with bound Eritoran was also considered [2Z56].

Model generation	Start position	MD-2 docking results
h LPS agx [3FXI]	---> h Lipid A agx3FXI = Lipid A agx	no bad vdW contacts
Lipid A agx [3FXI]	---> copy & extract Lipid IVA agx	no bad vdW contacts
Lipid A agi [3FXI]	---> copy & extract Lipid IVA anx	bad vdW contacts
Lipid IVA agx [3FXI]	---> copy & flip BB into Lipid IVA agi	no bad vdW contacts
Lipid IVA agi [3FXI]	---> copy & fuse FA4 into FA1 pose of Lipid A agi	no bad vdW contacts
Lipid IVA anx [2E59]	---> copy & extract Lipid IVA anx	no bad vdW contacts
Lipid IVA anx [2E59]	---> copy & flip BB into Lipid IVA agi (=Lipid A agx)	bad vdW contacts
Lipid IVA ani [2E59]	---> copy & fuse BB into Lipid A ani (=Lipid IVA anx)	bad vdW contacts

Only murine Lys367b can attract the phosphate group of the ligand. In the human, dog and horse systems, however, the equivalently positioned residues are repulsive anionic (acidic) residues (glutamate or aspartate). Moreover, in human, canine and equine species a conserved lysine functionally “neutralizes” aforementioned glutamate or aspartate residues by forming salt bridges (equine position 389b in Figure 3 in [6], Table 6 and 7). In mice, this position (equine 389b) is mSer386 (labeled 389b in Figure 1, Tables 6 and 7) leaving mLys367b active, i.e. a negatively charged side chain is absent and the cationic lysine cannot be neutralized. The sequence alignment study reveals that it is nonconserved (equine position 370b in Figure 1 or Figure 3 in [6], see also Tables 6 and 7). Hence in a unique fashion, it draws one backbone phosphate group into the agonist position (circle “Pag” in Figure 3 in [6]). Another nonconserved position on the outer lip of the MD-2 pocket entry is also involved (equine position 122c in Figure 1 here or Figure 3 in [6]). While positively charged lysine and arginine stabilize the presence of a phosphate group in close proximity to MD-2, there is a strong repulsion in presence of murine Glu122c. This favors phosphate repositioning – and with it the glucosamine backbone of Lipid IVA – into the agonist site within the wedge (circles “Pan” and “Pag” in Figure 3 in [6]).

**Table 6:** Listing differential residues on MD-2 regarding species-specific phosphate binding (chain c).

Homo sapiens	Mus	Equus
hLys58c	mAsn58c	eArg58c
hArg90c	mArg90c	eArg90c
hLys122c	mGlu122c	eArg122c
hAsp101c	mAsp101c	eAsp101c

**Table 7:** Differential or mechanistically relevant amino acids in species-specific binding to the phosphates of Lipid IVA.

Human TLR4	Murine TLR4	Equine TLR4
**hLys58c**	**mAsn58c**	**eGlu58c**
hGlu369b is reinforced by Asp371b repelling P2.	mLys367b remains attractive to P1 by Ala369b.	eGlu370b is neutralized in salt bridge with Lys372b.
No arginine, but Gly384a, cf. hGln436b.	mArg434b is equivalent to eArg385a to attract P1.	eArg385a is equivalent to mLys367b to attract P1
**hLys388b**	**mSer386b**	**eLys389b**
hLys388b is in salt bridges with Glu369b or Glu321a.	mSer386b has hydrogen-bond with Lys341a.	eLys389b forms a salt bridge with Glu344a.
hGly343a without interaction, but adjacent Glu321a is in a salt bridge with Arg322a.	mLys341a shifts from H-bond to form a stronger salt bridge with P.	eGlu344a is in salt bridge with Arg342a.
**hGln436b**	**mArg434b**	**eGln437b**
No cationic attraction to direct P2 into the agonist position.	Cationic mArg434b attracts P1 to direct the phosphate group into the agonist position.	Ridge of ion bridges and H-bonds: Glu394b+Lys389b Lys372b+Glu370b to ramp up P1 from the groove (TLR4*/MD-2 interface) to the wedge (TLR4/TLR4* interface)
No cationic attraction for P2 in wedge. P cannot mediate the TLR4*/MD-2 contact in the wedge, where it is attracted by hLys89c and hArg90c, disrupting the TLR4*/MD-2 interface near hGlu439b. Dimerization is not enabled.	mLys341a, mLys367b, mArg434b form cationic attraction for P1 in wedge. P1 can mediate the TLR4*/MD-2 contact in the wedge. The amide-bound fatty acid FA1 of Lipid IVA partially replaces FA1 of LPS in the TLR4*/MD-2 interface. Dimerization is established.	eLys366a, eArg385a, eLys389b attracts P1 in wedge. Only weakly, P1 mediates the TLR4*/MD-2 contact in the wedge since P1 is more attached to the TLR4 side than to TLR4*. The amide-bound fatty acid FA1 of Lipid IVA (in analogy to FA1 of LPS) may assist the TLR4*/MD-2 formation. Dimerization can be established.
Antagonist activity	Agonist activity	Partial agonist activity

**Figure 4:**
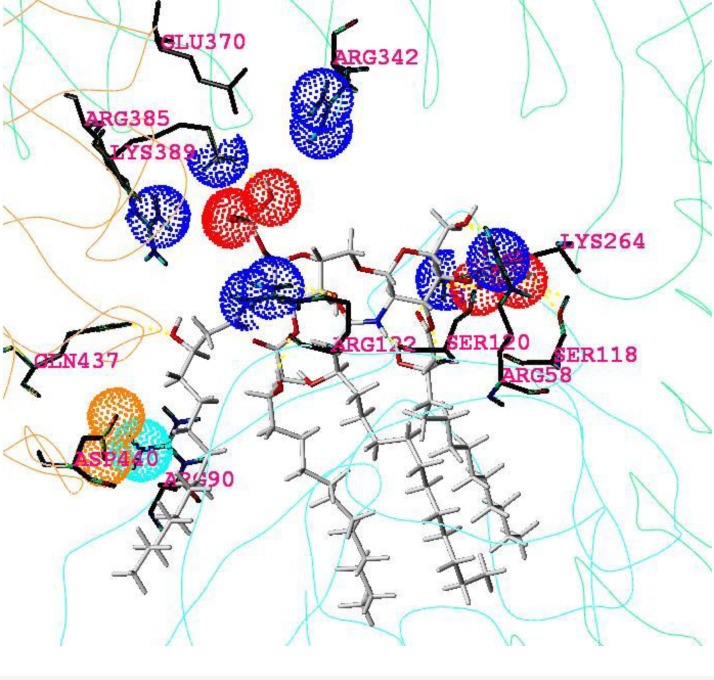
Docked agonist ligand Lipid IVA into the horse dimeric complex. Display and color code: light blue line: backbone of monomer unit (hTLR4 and hMD-2); sticks (Lipid IVA) in grey, red, and blue colors (atoms H, C, P, O, N, respectively); single or double blue dots mark terminal nitrogens of monocationic lysine, or arginine, respectively; triple red dots mark the three oxygen of monoanionic phosphate groups, double orange dots mark the two oxygen of glutamate anion on TLR4* and light blue double dots mark the two terminal nitrogens of monocationic arginine on MD-2. All carbon atoms of amino acids are colored in dark grey and hydrogen atoms are omitted. The three reported amino acids are colored with yellow heteroatoms [14]. Anionic Asp440b and cationic Arg90c enter into strong electrostatic attraction, forming an additional stabilizing element in the TLR4*/MD-2 interface, apparently, a prerequisite for dimerization and endotoxic signaling.

In close modeling to the murine and human complexes elucidated by x-ray crystallography (Figure 2 and Table 3 in [6] and [18, 19, 21, 22, 32]), a novel model of a equine TLR4/MD2/ligand complex has been generated. As shown in Figure 4, it proposes the *hitherto* unknown dimeric equine complex (eTLR4/eMD-2/Lipid IVA)_2_ with Lipid IVA in an agonistic binding orientation. The proposed interface between counterTLR4* (chain b) and MD-2 (chain c) of the monomeric subunit is found to be consolidated by a salt bridge formed between Asp440b and adjacent Arg90c that cannot be disrupted (labeled 440b in Figure 1 and labeled 90c in Figure 3, see also Tables 6 and 7). Most importantly, the phosphate group (P1) of Lipid IVA is bound in the agonist position by side chains of the nonconserved – and therefore horse-specific – residues Arg342a, Arg385b and Lys389b (Tables 6 and 7). In analogy to the murine system, it can be speculated that this may enable the protrusion of at least a significant aliphatic part of fatty acid FA1 into the TLR4*/MD-2 interface (analog of FA1 of LPS [18]) to stabilize this m-shaped dimerization complex by interaction with the reported hydrophobic patch exposed on TLR4* [39]. All told, the model suggests Lipid IVA acts as agonist in the equine TLR4/MD-2 system inducing receptor dimerization and signal transduction in the innate immune response of the horse.

### The modeled wedge between MD-2 and two TLR4 subunits

The general model of the complexes formed by MD-2 and TLR4 is that of a “wedge” (Figure 3 in [6]). The variable phosphate binding sites along the leftmost line of the wedge have been identified as the potential dimerization interface in the prior study of Meng *et al*. [14]. In a 2010 follow-up publication (with a wrong residue assignment in its title: Asp122 instead of Glu122), the interface of TLR4/MD-2 (without TLR4*) with hTyr42c (chain c is MD-2 in [18]) was mechanistically analyzed [14]. Hence, it can be assumed that stable heterodimers of TLR4 and MD-2 are present in an *a-priori* existing functional unit on the cell surface. Even though encoded by the corresponding individual genes, TLR4 and MD-2 may just be seen as two joint domains of a single ′fusion′ protein because they are coordinately expressed and the corresponding high-affinity complex has been shown to be pre-assembled in the trans-Golgi network during the vesicular transport of TLR4/MD-2 to the plasma membrane [19, 22, 42].

**Figure 5:**
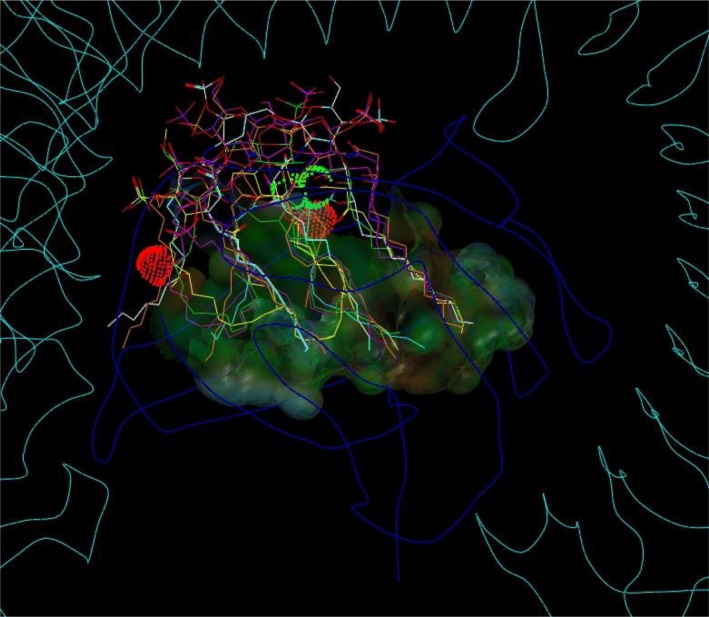
Superposition of observed and docked ligand poses into the “wedge” with the MD-2 pocket. The cavity surface appears translucent and color coded for hydrophilic (grey to bluish) and hydrophobic (green to brown) properties. Red or green labels and dots locate favorable (green) or unfavorable (red) interactions between the surrounding molecular components (Table 1). The backbones of MD-2 and both TLR4 are displayed as dark or light blue lines, respectively. The triangular interaction zone (“wedge”) is displayed (white dotted lines). The locations of the three clustered phosphate binding sites are labeled accordingly: TLR4*-attractive “Pag” (green), TLR4-attractive “Pag-Pan” (green), or TLR*-repulsive “Pan” (red). Note, the red dots and the red “Pan” label (to the left) indicate that the counter TLR4* is rejected while TLR4 stays complexed to MD-2 (green labels). The rightmost corner (TLR4-attractive “Pag-Pan”) corresponds to the highly conserved phosphate binding site, common to all ligands regardless of agonistic or antagonistic activities (right corner in Figure 3 in [6]). The labels “F” locate TLR4 / MD-2 interFaces and their colors indicate the ligand-dependent type of interaction: red “F” for repulsion caused by liganded antagonists only, and only green “F” for both, agonists and antagonists.

The hydrophobic cleft of MD-2 displays a characteristic asymmetric bi-partite composition: The first, ′lower right′ part of the MD-2 cavity is located at the ′primary′(*a priori*) complexation site between MD-2 and TLR4 and contains the deepest and most hydrophobic part of the groove accommodating the two (R)-3-acyloxyacyl units, i.e. fatty acids FA3, 3’, 4 and 4’ attached to the GlcN II moiety of LPS/lipid A. The second major part of the cleft is located in the opposite direction at the left ′opened′ side near to the ′secondary′ dimerization zone in the wedge model and represents a distinct more hydrophilic substructure within the MD-2 cavity. This second major subsite of the MD-2 cleft confers the protrusion of the amide-bound ′primary′ 3-hyxdroxymyristoyl residue FA1 - also designated as acyl residue “A” in the literature - attached to the GlcN I moiety of LPS/lipid A (Figure 3). Since hydrophobic burying of an alkyl chain is energetically disfavored in this zone (left side of MD-2) this fatty acid chain can apparently protrude without significant energetic difference; i.e. partial solvent exposition of FA1 (A) does not appear to be more destabilizing than burying it in a nonpolar manner within the TLR4/MD-2 single subunit complex. Apparently, in presence of another subunit (counter TLR4*/MD-2*) the protrusion of this acyl residue becomes even the preferred action. Actually, upon the assembly of the active receptor complex protruding and interacting with the hydrophobic patch zone on TLR4* represents an optimal way of minimizing the repulsive forces between this alkyl chain and the surrounding water shell, i.e. “burying” it very efficiently in the extended hydrophobic pocket formed by TLR4/MD-2 and counter TLR4*, thus forming the bridging interface between TLR4/MD-2 and counter TLR4* (left side of the wedge in Figure 5).

### Mapping the binding relevant residues to determine species dependencies

**Figure 6:**
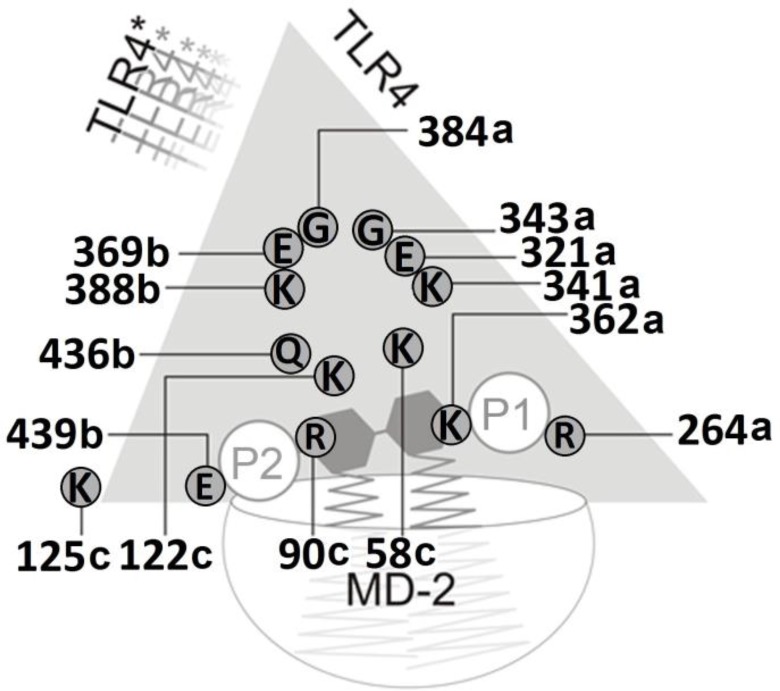
Schematic view of the computed side chain interactions of the complete human TLR4 ectodomain/MD-2 complex with Lipid IVA pointing out the receptor antagonistic action of Lipid IVA in the human system. Note the inverted (flipped) orientation of the sugar-phosphate backbone as compared to the agonistic binding of lipid A/LPS to human TLR4/MD-2 as well as to the agonistic interaction of Lipid IVA with murine TLR4/MD-2 (see Fig. 7). The indicated charged/polar side chains of counter-TLR4* form a “repulsion region” that prevents the association of the TLR4/MD-2/Lipid IVA unit in the human system. As a direct consequence Lipid IVA does not provide the dimerization into an active m-shaped receptor complex in the human system and acts as a competitive inibitor of LPS/lipid A (agonist) binding instead. Counter-subunit TLR4* is leaving or never was in place, which is indicated by the shaded label of TLR4* (upper left side of wedge).

**Figure 7:**
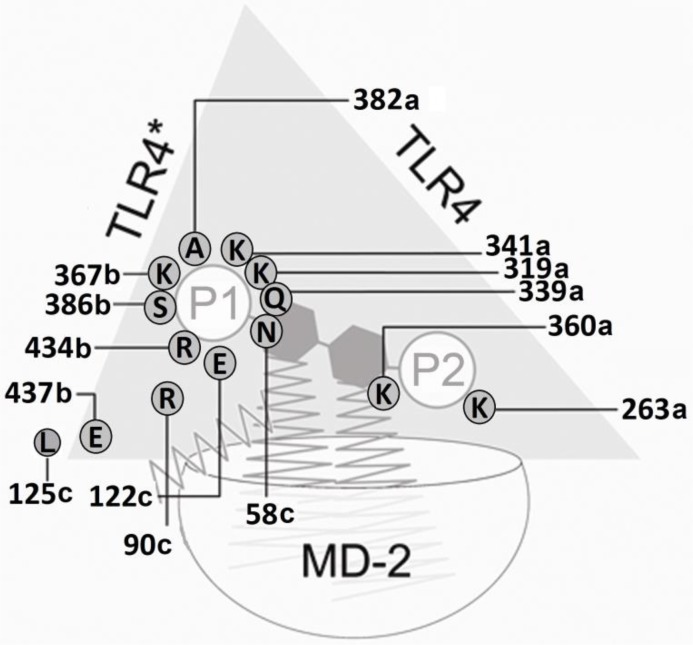
Schematic view of the computed side chain interactions of the murine TLR4 ectodomain/MD-2 complex with Lipid IVA pointing out the agonistic action of Lipid IVA in the mouse system. The repulsive, anionic sidechain interaction of a glutamate residue specifically present in murine MD-2 (Glu122c) favors the upward shift of the phosphate residue P1 (1-PO_4_) at GlcN-I into the bridging position formed by a cluster of residues of TLR4, MD-2 and TLR4*. In line with the current *consensus* model of agonist-induced TLR4/MD-2 activation dimerization into the active m-shaped receptor complex takes place in consequence.

**Figure 8:**
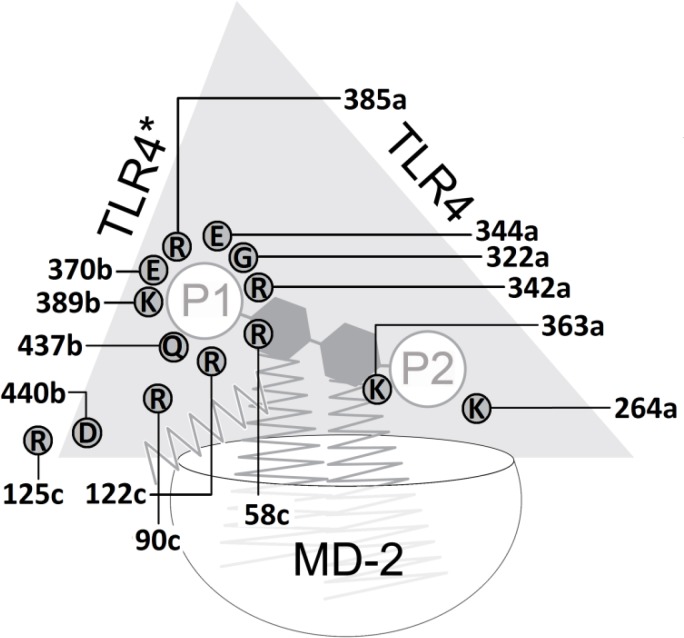
Schematic view of the computed side chain interactions of the equine TLR4 ectodomain/MD-2 complex with Lipid IVA indicating the (weakly) agonistic action of Lipid IVA in the horse system. The phosphate group P1 of the ligand connects the equine TLR4/MD-2 unit with its counter-TLR4*. The eMD-2 vestibule is as rich in cationic residues as the human one (Figure 6), what reflects the weak agonism in horse. Mainly attractive forces of chain “a” arginine (Arg385a) assist the agonistic position (left side, phosphate P1 attached to glucosamine GlcN-1) with its dimerization propensity.

Inspecting the results of the x-ray structures and modeling-based three-dimensional mapping of the species differences in the wedge reveals, that only the murine TLR4/MD-2 system possesses two attractive (cationic) side chains: lysine and arginine (mLys367b, mArg434b (labels 370b and 437b in Figure 1 , also Figures 6, 7 and 8). For this reason in mice, the attraction of the anionic charge of that phosphate group is stronger than in the other mammalian species compared here. For a ligand (Lig) to become an agonist, the occupation of the uppermost phosphate patch which constitutes a more TLR4*-exposed area, enables bridging both monomers (TLR4/MD-2/Lig and TLR4*/MD-2*/Lig*) to form an active dimeric subunit complex (TLR4/MD-2/Lig)_2_. In addition, in the mouse system a lysine side chain (mLys319a, not mLys341a) clearly contributes to the phosphate binding of the ligand in the agonist position (labels 322a and 344a in Figure 1). The phosphate binding site pattern in the wedge zone of the mouse differs mainly from that of the other three species because the phosphate is attracted by mLys367b whilst the analog horse lysine of counter-TLR4* on chain b (eLys389b) is neutralized in a salt bridge. As a sort of compensation the ligand moves upward under the influence of the very long and branched side chain of eArg90c.

### Interpreting the concert of mechanistically relevant residues identified by docking and mapping studies

Concerning agonistic Lipid IVA, in the murine MD-2 acidic mGlu122c causes electrostatic repulsion of the monoanionic phosphate group which moves up (to “Pag” in Figure 3 in [6]) while the corresponding *homo* and *equus* residues 58 and 122 on strand 3 of sheet “C” (hLys58c, eArg58c, hLys122c, eArg122c, see also Tables 6 and 7) exercise attraction forces on phosphate keeping the latter in closer position to the antagonistic site. The cationic side chains of eArg58c and eArg122c are longer than hLys58c and hLys122c which reflects that ligand Lipid IVA in human systems lies in antagonistic pose with its phosphate group (“P2” in Figure 6) located in the lower left corner of the wedge (“Pan” in Figure 3 in [6]) whilst Lipid IVA in equine systems (partial or weak agonism) can move into a “in-between” position, that is between human and mouse poses in the upper left and lower left corner of the wedge, respectively (Figure 3 in [6], also Figures 4, 5 and 8).

Additional ligand-relevant amino acids of MD-2 were also inspected and listed (Tables 6 and 7). The group of mechanistically interacting side chains lies on all sides of the wedge between TLR4, TLR4* and MD-2 (Table 7).

Intriguingly, Phe126 of human MD-2 (strand 5 of sheet “B”) interacts with TLR4*/MD-2 and was reported by Park *et al*.: “The phenylalanine 126 loop undergoes localized structural change and supports this core hydrophobic interface by making hydrophilic interactions with TLR4” [18]. In full agreement with Meng *et al*. our binding model also predicts the phosphate attracting role of cationic residues in position 122 on human and equine chains c (MD-2), whereas glutamate 122c assists the upward shift of the phosphate group (Figure 3 in [6] as well as Figure 2) [14]. The listed results extend Meng's report (Tables 6 and 7) [14].

## Conclusions

The binding modes were established based on experimental observations in the literature and some of the identified residues had been reported as species-relevant in earlier mutagenesis studies. In contrast to the major natural ligand ′hexa-acyl type′ lipid A present in LPS of various commensal and pathogenic Gram-negative bacteria, ′under-acylated′ precursor or synthetic structures such as Lipid IVA or Eritoran represent much more non-natural congeners and thus particular ′imperfect′ ligands of mammalian TLR4/MD-2. With respect to the human receptor system, both of latter tetra-acylated compounds cannot connect TLR4 with counter TLR4* through their flipped biphosphorylated diglucosamine backbone and are more deeply buried in the MD-2 cavity by about 4 to 5 Å with respect to LPS. As described here more in detail for comparison of the human, murine and equine TLR4/MD2 complexes, it is save to conclude that Lipid IVA constitutes rather an “accidental” agonist or antagonist (lower affinity ligand) than an agent with dual activity. As an agonist it can bridge the gap between the phosphate binding site of TLR4/MD-2 and the counter unit TLR4”. As a flipped antagonist it is more deeply buried into MD-2. Only one phosphate group (P1 of glucosamine GlcN-1) can occupy the more conserved phosphate binding location in the lower right corner of the wedge. The other phosphate group (P2 of GlcN-2), however, cannot reach the phosphate binding site of agonists like LPS, which is composed of side chains of counter TLR4* and one variable TLR4 residue. Dimerization is then triggered by connecting a TLR4/MD-2 unit to the counter TLR4*. Agonistic activity is always associated with ligand-brigded dimerization of the TLR4/MD-2 complex. The phosphate binding residues between TLR4 and MD-2 in the rightmost corner of the wedge model are highly conserved. The conservation reflects the need of preserving an optimal grip (attraction) on one phosphate group as a “primary” or imperative fixation point because its binding always takes place, regardless the agonistic or antagonistic nature of the ligands. In contrast, the other phosphate location varies due to species-specific changes in the side chains from both sides (TLR4, TLR4*) of the wedge. Upon their binding agonists lead to a TLR4/MD-2/Lig dimerization in a defined m-shaped complex triggering the recruitment of adapter proteins to the cytolpasmic TIR domains of the TLR4 subunits and in turn the activation of multiple downstream-signaling cascades which provide the pro-inflammatory response. The results were unambiguously confirmed by newer crystal structures in 2012 (PDB codes, 3VQ1 and 3VQ2).

## Glossary and Terminology

/: Interface symbol linking protein subunits TLR4, TLR4*, MD-2 or MD-2*
ABCD: Chain A = TLR4, B = TLR4*, C = MD-2, and D = MD-2*, see [18]
flipped: 180° rotation of glucosamine backbone of Lipid IVA or Eritoran compared to LPS
GlcN: glucosamine(s) of the diglucosamine backbone of LPS
MD-2: Myeloid differentiation factor 2, lymphocyte antigen 96
TLR4: Toll-like receptor 4, CD284 antigen
Wedge: Triangular empty space with three interfaces on each side: TLR4 - MD-2, TLR4 - TLR4* and TLR4*/MD-2
